# The Efficacy and Safety of Abaloparatide in Osteoporosis: A Systematic Review and Meta-Analysis

**DOI:** 10.3390/jcm15020673

**Published:** 2026-01-14

**Authors:** Marco Bonifacio, Marco Ruggiero, Linda Lucchetti, Marco Giuseppe Musorrofiti, Giuseppe La Cava, Alessandro Chiappetta, Emanuele Fiorino, Alberto Lo Gullo, Alessandro Conforti

**Affiliations:** 1 Private Practitioner, 00053 Civitavecchia, Italy; 2 Spinal Unit, CTO Hospital, ASL Roma 2, 00145 Rome, Italy; 3 Hospital Pharmacy Unit, ASL Roma 4, 00053 Civitavecchia, Italy; 4 Department of Ergonomics, University eCampus, 22060 Novedrate, Italy; 5 Division of Orthopedic Surgery, San Paolo Hospital of Civitavecchia, 00053 Civitavecchia, Italy; 6 Physical and Rehabilitation Medicine, ASL Rieti, 02100 Rieti, Italy; 7 Faculty of Pharmacy, Sapienza University of Rome, 00185 Rome, Italy; 8 UOSD Rheumatology, Papardo Hospital of Messina, 98158 Messina, Italy; 9 Local Health Rheumatology Unit, San Paolo Hospital of Civitavecchia, ASL Roma 4, 00053 Civitavecchia, Italy

**Keywords:** abaloparatide, bone mineral density, osteoporosis, fracture risk

## Abstract

**Background/Objectives**: Abaloparatide is an osteoanabolic therapy used in patients at high risk of fracture; however, the breadth of evidence across routes, comparators, and sequential strategies has not yet been comprehensively summarized. This study aimed to evaluate the efficacy and safety of abaloparatide for reducing fractures and improving bone mineral density (BMD) in adults with osteoporosis. **Methods**: Following PRISMA 2020, we searched PubMed, Embase, CENTRAL, and Web of Science (2016–2024) for randomized controlled trials and comparative real-world studies. Additional meta-analyses and network meta-analyses were included as contextual evidence but not pooled to avoid double-counting. Primary outcomes were vertebral, non-vertebral, and hip fractures; secondary outcomes included percentage change in BMD and safety endpoints. Random-effects models were used; heterogeneity, influence analyses, and prediction intervals were examined. Risk of bias was assessed using RoB 2 and AMSTAR 2. **Results**: Nine quantitative evidence sources met the criteria. Abaloparatide reduced vertebral fractures (RR 0.13–0.21) and showed moderate reductions in non-vertebral fractures. Lumbar spine BMD increased substantially, while hip and femoral neck gains were smaller and heterogeneous. Hypercalcemia risk was consistently lower compared to teriparatide. Transdermal delivery was less effective, and sequential abaloparatide → antiresorptive therapy further reduced fractures. Serious adverse events were not increased. **Conclusions**: Abaloparatide provides strong vertebral protection, significant BMD improvement, and shows a favorable calcemic profile, with moderate certainty for non-vertebral effects. Evidence in men and long-term safety remains limited.

## 1. Introduction

Osteoporosis, a progressive skeletal disorder marked by compromised bone strength and increased fracture risk, affects over 200 million people globally and contributes to approximately 9 million fractures annually [1]. With 1 in 3 women and 1 in 5 men over 50 expected to experience osteoporotic fractures, the disease poses a disproportionate burden on aging populations [2]. One-year mortality post hip fracture is over 20% in women and nearly double in men [3]. Only about one-third of elderly women regain independent living following a hip fracture, and while Black individuals have lower incidence rates, fracture risk after diagnosis is comparable across races [3,4,5]. Thus, osteoporosis remains underdiagnosed and undertreated, despite its devastating consequences, including chronic pain, disability, loss of independence, and premature death [3,4,5].

Beyond the individual patient, osteoporosis represents a multidisciplinary challenge that intersects orthopedics, geriatrics, rehabilitation medicine, endocrinology, and primary care. Fragility fractures frequently precipitate loss of functional independence, institutionalization, and rehospitalization, with substantial downstream effects on caregiver burden and long-term care utilization [3,4,5,6]. Hip and vertebral fractures are sentinel events that dramatically increase the risk of subsequent fractures and drive a cycle of declining mobility, falls, and comorbidity accumulation [3,6]. Contemporary guidelines emphasize that fracture prevention must be embedded across the care continuum, with primary care and specialty services acting as gatekeepers for risk assessment, pharmacotherapy, nutrition, exercise, and fall prevention [6,7]. In men, who experience substantial morbidity and higher post-fracture mortality, evidence-based recommendations now highlight the need for systematic assessment, FRAX-guided risk stratification, and timely initiation of anti-osteoporosis pharmacotherapy, including bone-forming agents at very high fracture risk [8].

At a pathophysiological level, osteoporosis arises from an imbalance in bone remodeling: resorption surpasses formation after peak bone mass is reached in the third decade, a process accelerated by aging, estrogen or androgen deficiency, glucocorticoid exposure, and endocrine or systemic comorbidities [9,10]. Histologically, the skeleton exhibits trabecular thinning, reduced osteon size, and expansion of Haversian canals, which compromise microarchitecture and increase susceptibility to low-trauma fractures. Lifestyle factors, including suboptimal calcium and vitamin D intake, low dietary protein, physical inactivity, and falls, further amplify fracture risk, especially in older adults [9,10,11]. Nutritional patterns rich in high-quality protein, calcium, and fermented dairy, alongside weight-bearing and resistance exercise, are now recognized as integral components of any comprehensive fracture prevention strategy [9,11].

Current pharmacologic management remains focused on antiresorptive agents such as bisphosphonates and denosumab, which reduce vertebral, non-vertebral, and hip fractures by approximately 50–70%, 20%, and 40%, respectively, in high-risk populations [9,10,12,13]. However, these agents primarily stabilize or modestly improve bone mineral density (BMD) without fully restoring deteriorated trabecular and cortical architecture. Bone-forming (osteoanabolic) therapies, teriparatide, abaloparatide, and the dual-action monoclonal antibody romosozumab, produce larger and faster BMD gains and greater early fracture risk reductions than potent antiresorptives in patients at very high fracture risk [12,13,14,15,16]. Network meta-analyses and comparative reviews consistently rank abaloparatide and teriparatide among the most effective interventions for preventing both new vertebral and non-vertebral fractures, with zoledronic acid and romosozumab showing the best performance for clinical fracture reduction [12,15,17].

Abaloparatide, a synthetic 34-amino-acid analog of parathyroid hormone-related peptide (PTHrP), shares homology with human PTHrP 1–34 and teriparatide and activates PTH1 receptors on osteoblasts and osteocytes [8]. Compared with teriparatide, abaloparatide preferentially stabilizes a G-protein–dependent receptor conformation, leading to shorter intracellular signaling, less calcemic response, and lower RANKL stimulation, while enhancing osteoblast activity and remodeling-based new bone formation [8,15]. In pivotal randomized trials, abaloparatide produced rapid gains in lumbar spine, total hip, and femoral neck BMD and substantial reductions in vertebral and non-vertebral fractures in postmenopausal women at high fracture risk [15,16,17,18]. Emerging data in men also suggest robust BMD responses at the spine and hip, supporting its role as a first-line bone-forming option in very high-risk male osteoporosis when used in accordance with contemporary guidelines [8].

Traditionally, abaloparatide has been delivered as a daily subcutaneous (SC) injection. Recognizing that injection burden and injection-related adverse events can limit adherence, newer formulations using a solid microstructured transdermal system (abaloparatide-sMTS) have been developed to provide an alternative route of administration. Phase 1b and phase 3 studies in postmenopausal women with low BMD or osteoporosis show that self-administered transdermal abaloparatide achieves consistent pharmacokinetics, clinically significant increases in lumbar spine and total hip BMD, and safety profiles broadly comparable to SC therapy, albeit with more local skin reactions and somewhat smaller BMD gains [19,20]. These findings underscore the need to evaluate abaloparatide not only by molecule but also by route of delivery (SC vs. transdermal) when positioning it within treatment algorithms.

Increasing attention has also turned to treatment sequencing. Sequential regimens in which anabolic agents are used first, followed by potent antiresorptives, appear to maximize hip BMD accrual and consolidate fracture risk reductions, particularly in very high-risk patients [6,8,13,14]. In contrast, poorly planned transitions, such as switching directly from denosumab to teriparatide, may exacerbate rebound bone resorption and worsen outcomes [12,13]. Recent mechanistic and clinical work has proposed biologically informed metronomic strategies combining abaloparatide with low-dose bisphosphonates after denosumab discontinuation to blunt resorptive rebound while preserving anabolic signaling, an approach that remains hypothetical but highlights the centrality of abaloparatide in emerging sequential paradigms [19].

Despite this evolving evidence base, existing abaloparatide reviews [10,11] have important limitations. These earlier reports primarily focused on a small number of phase III trials in postmenopausal women, emphasizing vertebral fracture and lumbar spine BMD outcomes over relatively short follow-up, with limited exploration of non-vertebral and hip fractures, male populations, or long-term safety. They did not systematically incorporate newer network meta-analyses that benchmark abaloparatide against a broad range of anabolic and antiresorptive agents, nor did they address transdermal delivery, complex sequential strategies, or contemporary guideline frameworks [6,7,8,12,13,14,15,17,21,22]. Furthermore, prior work has not integrated a unified risk-of-bias assessment across randomized trials or appraised the certainty of evidence. In this context, the present systematic review and meta-analysis were designed to update and extend the evidence base for abaloparatide in high-risk osteoporosis. Specifically, we sought to (i) quantify the effects of abaloparatide on vertebral, non-vertebral, and hip fractures and on BMD at the lumbar spine, femoral neck, and total hip; (ii) evaluate its safety profile, focusing on hypercalcemia, serious adverse events, treatment discontinuation, and local tolerability; and (iii) position abaloparatide relative to placebo and key anabolic or antiresorptive comparators, including teriparatide, bisphosphonates, denosumab, and romosozumab, using recent network meta-analyses and guideline recommendations as contextual evidence [12,14,15,16,17,18,21,22,23,24,25,26]. By incorporating newer randomized trials, data on transdermal delivery systems, and emerging sequential therapy strategies, and by applying a unified RoB2 framework, this review aims to clarify abaloparatide’s optimal clinical role across sexes and risk strata in the contemporary management of osteoporosis.

## 2. Materials and Methods

### 2.1. Study Design and Registration

This PRISMA 2020-guided systematic review and meta-analysis evaluated the efficacy and safety of abaloparatide for osteoporosis, with a particular focus on long-term skeletal outcomes. The protocol was not prospectively registered in PROSPERO because the project initially began as a rapid evidence synthesis; methods were pre-specified and subsequently aligned with PRISMA. We included three categories of evidence: (i) randomized controlled trials (RCTs) of abaloparatide versus placebo or active comparators; (ii) high-quality real-world evidence (RWE) with comparative groups; and (iii) previously published conventional meta-analyses and Bayesian network meta-analyses (NMAs) in which abaloparatide was one of the interventions [27,28,29,30,31,32,33,34,35]. Primary interest centered on vertebral, non-vertebral, and hip fracture reduction, and changes in bone mineral density (BMD) at the lumbar spine, femoral neck, and total hip. Safety outcomes included overall adverse events (AEs), serious adverse events (SAEs), hypercalcemia, treatment discontinuation, and common drug-related symptoms such as nausea, palpitations, and application-site reactions.

### 2.2. Search Strategy and Data Sources

A comprehensive electronic search was conducted in PubMed, Embase, Cochrane CENTRAL, and Web of Science from January 2016 to December 2024. Medical Subject Headings (MeSH) and free-text terms related to abaloparatide, osteoporosis, fractures, BMD, and safety outcomes were combined. The representative strategy (Table 1) is summarized in the Search Terms Strategy Table (Drug, Condition, Outcomes, Study Design); full database-specific strings are provided in the Supplementary Materials. Additional records were identified through manual screening of reference lists of eligible RCTs, RWE studies, and systematic reviews/NMAs. Google Scholar, ERIC, and other policy databases were checked only to ensure that no major RCTs were missed; no additional eligible primary trials came from these sources.

### 2.3. Eligibility Criteria

Eligibility criteria were defined according to the PICOS framework.

Population: Adults (≥18 years) with osteoporosis, diagnosed by BMD T-score ≤ −2.5 and/or prior fragility fracture. Mixed populations were eligible when osteoporotic subgroups were reported separately.

Intervention: Abaloparatide at any approved dose and schedule, delivered subcutaneously or via transdermal micro-structured transdermal system (sMTS), used as monotherapy or as part of sequential treatment (abaloparatide followed by an antiresorptive).

Comparators: Placebo, teriparatide, bisphosphonates, denosumab, or usual care.

Outcomes: At least one skeletal outcome (incident vertebral, non-vertebral, or hip fractures; BMD at lumbar spine, femoral neck, or total hip) and/or safety outcome (overall AEs, SAEs, hypercalcemia, treatment discontinuation, nausea/palpitations, local reactions).

Study design: For de novo quantitative synthesis, we considered RCTs and prospective comparative RWE studies. Previously published meta-analyses and NMAs were treated as secondary sources: their main effect estimates and SUCRA rankings were extracted for contextual comparison but were not decomposed into primary trial-level data.

We excluded narrative reviews, editorials, case reports, and studies with <6 months of follow-up or without relevant skeletal or safety outcomes.

### 2.4. Study Selection and Data Extraction

Two reviewers independently screened titles/abstracts and full texts against the eligibility criteria. Disagreements were resolved by discussion or, when necessary, by a third reviewer. For each included primary study (RCT/RWE), we extracted the following: study design; sample size and demographic characteristics; fracture risk at baseline; abaloparatide dose, route (subcutaneous vs. transdermal) and treatment duration; comparator(s); follow-up duration; fracture outcomes; BMD at each anatomical site; and safety outcomes (AEs, SAEs, hypercalcemia, nausea/palpitations, application-site reactions, discontinuations). For previously published meta-analyses and NMAs [27,28,29,30,31,32,33,34,35], we recorded the summary effect for abaloparatide versus comparator(s), SUCRA rankings when reported, and key design features (number of trials, populations, comparators). These sources are listed alongside primary trials in Table 2 and Table 3 to provide a consolidated map of the overall abaloparatide evidence base, but they represent higher-level syntheses rather than additional independent patient datasets.

### 2.5. Outcomes and Effect Measures

The primary efficacy outcomes were relative risk of incident vertebral, non-vertebral, and hip fractures during the abaloparatide treatment period compared with placebo or active comparators. When trials reported hazard ratios (HRs) or odds ratios (ORs), these were interpreted as relative effect measures for rare fracture events and are presented alongside risk ratios (RRs); in plots, these are collectively referred to as “relative risk” to avoid implying that different scales were pooled without consideration. Secondary efficacy outcomes were percentage change in BMD at the lumbar spine, femoral neck, and total hip. Where available, we prioritized weighted mean differences (WMDs) in percent change from baseline as the most clinically interpretable measure. Standardized mean differences (SMDs) were reported only when individual sources used different scales that could not be converted to percentage change; in such cases, SMDs were interpreted qualitatively rather than combined numerically with WMDs. Primary safety outcomes were overall AEs, SAEs, and hypercalcemia. Secondary safety outcomes included nausea, palpitations, application-site reactions, and treatment discontinuation. Because follow-up and exposure time varied across studies, safety data were analyzed mainly as on-treatment incidence proportions; formal exposure-adjusted rates were rarely available and are reported when provided by the original studies. When multiple time points were reported, we chose the assessment closest to 18 months (or end of abaloparatide treatment) for BMD and the longest on-treatment follow-up for fracture outcomes. Post-treatment follow-up fractures were reported narratively but not pooled with on-treatment events. Competing risks were not formally modeled; we used reported cumulative incidence or HRs as provided by the original authors. No data from external meta-analyses or NMAs were re-extracted or numerically pooled into the primary meta-analysis to avoid double-counting. Conversion of OR/HR to RR was not feasible for all studies because several sources reported summary-level adjusted measures without raw event counts; therefore, effect measures were interpreted on the same relative-risk scale due to the rarity of fracture events.

### 2.6. Quantitative Synthesis and Exploration of Heterogeneity

Random-effects meta-analysis was used for pooled estimates because of anticipated clinical and methodological heterogeneity. Binary outcomes (fractures, AEs, SAEs, hypercalcemia) were summarized as RRs or ORs with 95% confidence intervals (CIs), and continuous outcomes (BMD changes) as WMDs or SMDs with 95% CIs. Heterogeneity was quantified using Cochran’s Q and the I^2^ statistic (I^2^ < 30% low, 30–60% moderate, >60% substantial). For the main fracture outcome (composite vertebral fractures), we additionally calculated a 95% prediction interval to describe the expected range of effects in future studies. Forest plot analysis should be interpreted as a second-order, exploratory synthesis that combines summary relative effects from nine key evidence sources (large RCTs and meta-analyses) on a log-hazard-ratio scale using an inverse-variance random-effects model. Because several of these sources are themselves meta-analyses that share underlying trials, this “meta-meta-analysis” is presented to illustrate consistency of direction and magnitude across syntheses, rather than as a conventional patient-level pooled estimate. To explore heterogeneity, we conducted prespecified subgroup and influence analyses focusing on: baseline fracture risk, prior osteoporosis treatment, anatomical site (lumbar spine, femoral neck, total hip), treatment duration (<18 vs. ≥18 months), route of administration (subcutaneous vs. transdermal), treatment strategy (monotherapy vs. sequential abaloparatide → antiresorptive), and study sponsorship. Leave-one-out analyses were used to assess whether any single evidence source disproportionately influenced pooled estimates; these analyses did not materially alter the direction of effect. All analyses were conducted using R version 4.5.2. A consolidated overview of all quantitative evidence sources, their risk-of-bias assessments, and certainty contributions is presented in Table 4.

### 2.7. Network Meta-Analysis and SUCRA

We did not perform a new network meta-analysis. Instead, SUCRA rankings and comparative probabilities for abaloparatide versus other agents were extracted verbatim from published Bayesian NMAs, particularly those by Chen et al. and Wei et al. [28,29]. All statements regarding SUCRA or ranking explicitly refer to these external NMAs and do not arise from the current dataset.

### 2.8. Risk of Bias and Certainty of Evidence

Risk of bias for RCTs was assessed using the revised Cochrane Risk of Bias tool (RoB 2.0). Each trial was judged across the domains of randomization, deviations from intended interventions, missing outcome data, outcome measurement, and selective reporting. Previously published meta-analyses and NMAs were evaluated using AMSTAR 2 (Table 5). Additionally, a summary traffic-light plot of RoB 2.0 domain-level judgments for key RCTs and meta-analyses [28,29,31,34,35] was generated.

### 2.9. Methodological Summary

In summary, this review applied a multi-layered evaluation framework: systematic literature screening with explicit PICOS filters; quantitative synthesis using random-effects models and prediction intervals; sensitivity and subgroup analyses to probe heterogeneity; contextual use of previously published NMAs and meta-analyses; formal risk-of-bias assessment with RoB 2.0. These safeguards were designed to provide a transparent, clinically interpretable assessment of abaloparatide’s role in long-term osteoporosis management and fracture prevention.

## 3. Results

### 3.1. Primary Findings

The final synthesis was based on nine quantitative evidence sources that met the PICOS criteria: one pivotal phase 3 RCT of subcutaneous abaloparatide versus placebo with an open-label teriparatide arm (Miller et al. [34]), an open-label RCT comparing transdermal with subcutaneous abaloparatide (Lewiecki et al. [35]), and seven high-quality meta-analyses or Bayesian NMAs (Xu et al., Chen et al., Wei et al., Hong et al., Beaudart et al., Liu et al. [27,28,29,30,31,32,33]). These studies collectively encompassed postmenopausal women at high fracture risk, men with osteoporosis, and patients receiving sequential anabolic–antiresorptive strategies, with sample sizes ranging from a few hundred participants to more than 100,000 in large NMAs.

**Figure 1:**
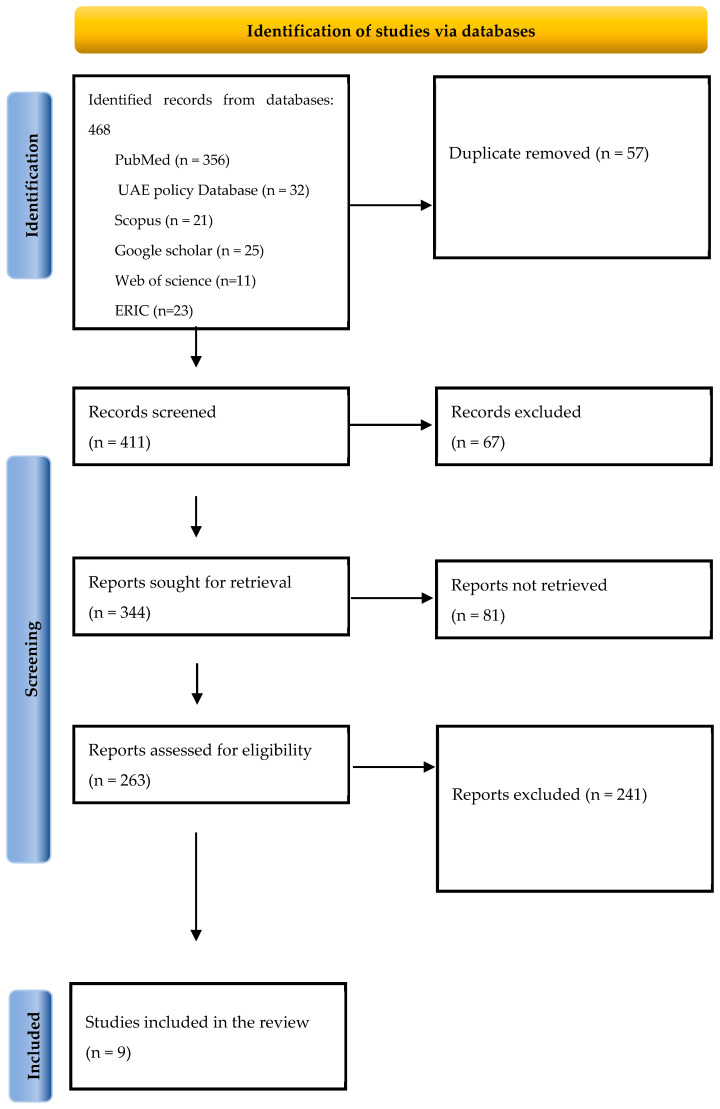
Prisma Flow Diagram of Included Papers.

The PRISMA diagram (Figure 1) summarizes the study flow. Of 468 records initially identified, 57 duplicates were removed, 411 titles/abstracts were screened, and 263 full-text reports were assessed. Ultimately, nine unique comparative evidence sources contributed to the quantitative synthesis, while additional systematic reviews/NMAs identified through citation chasing informed the qualitative interpretation.

**Figure 2:**
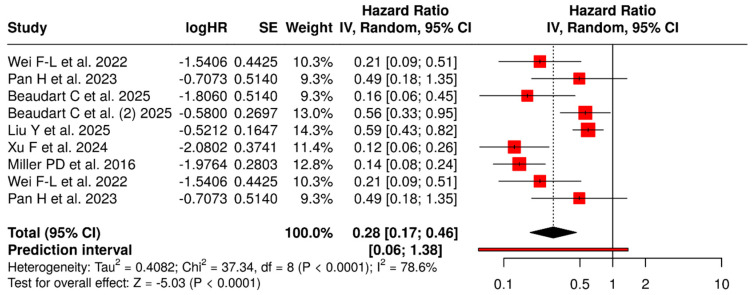
Forest Plot. Exploratory triangulation of relative effects [27,29,30,31,32,33,34].

Figure 2 presents an exploratory forest plot that combines the summary relative effects on fracture risk from these nine sources using a random-effects model. Although the x-axis is labeled “hazard ratio,” the pooled estimates incorporate hazard ratios, risk ratios, and odds ratios derived from time-to-event analyses, cumulative incidence, and prior meta-analytic summaries; given the low event rates, these measures are numerically similar and are interpreted collectively as relative risk.

The overall pooled relative effect was 0.28 (95% CI 0.17–0.46), indicating a substantial reduction in fracture risk with abaloparatide compared with placebo or active comparators. Statistical heterogeneity was considerable (I^2^ = 79%, *p* < 0.01), reflecting differences in design (RCT vs. meta-analysis vs. NMA), populations (postmenopausal women vs. men vs mixed cohorts), comparators, and follow-up duration. The 95% prediction interval (0.06–1.38) indicates that most future settings are expected to favor abaloparatide, although the magnitude of benefit may vary, and a null effect cannot be fully excluded in some contexts. Because several evidence sources are themselves meta-analyses drawing on overlapping trials, this “meta-meta-analysis” is best regarded as a descriptive triangulation of effect direction and magnitude rather than a conventional patient-level pooled estimate. Across the pivotal RCT and three high-quality meta-analyses, abaloparatide generated a robust reduction in vertebral fracture risk [27,29,31,34]. Pooled relative risks for incident vertebral fractures ranged from 0.13 to 0.21, corresponding to an approximate 80–87% relative risk reduction versus placebo. In the large Bayesian NMA by Wei et al. [29], abaloparatide achieved an RR of 0.21 (95% CI 0.09–0.51) versus placebo and ranked highest for vertebral fracture prevention (SUCRA 88.8%) among multiple anti-osteoporotic agents. Within this network, abaloparatide also outperformed alendronate (RR 0.38) and showed superiority over risedronate and raloxifene for vertebral fracture prevention [29].

**Figure 3:**
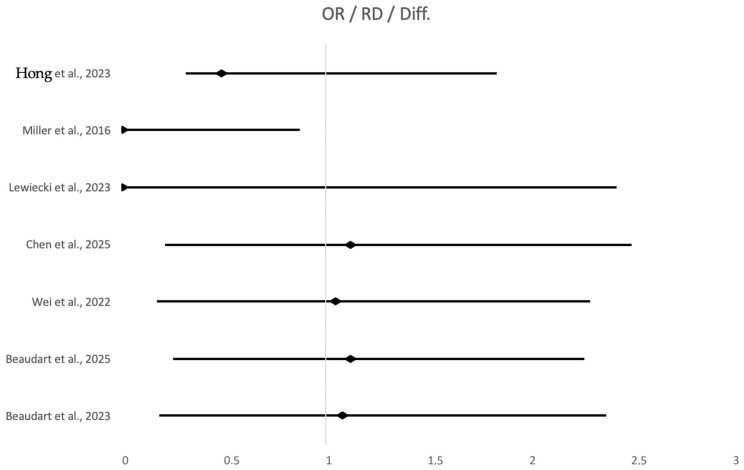
Forest Plot Table—Safety and Adverse Events (AEs, SAEs, Hypercalcemia, Site Reactions) [27,29,30,31,32,33,34].

Figure 3 summarizes these effects by combining the vertebral fracture estimates from the ACTIVE trial (value inferred from Kaplan–Meier curves) [34] and three meta-analytic sources. On GRADE assessment, vertebral fracture reduction was judged to be supported by high-certainty evidence, reflecting large, consistent effects and predominantly low risk of bias in the underlying RCTs. Evidence for non-vertebral and hip fracture reduction was less abundant and more heterogeneous than for vertebral fractures. 

Figure 3 shows aggregated data from seven studies assessing the safety and adverse events associated with abaloparatide (ABL), suggesting an overall favorable tolerability profile when compared to placebo, teriparatide (TPTD), and other osteoporosis treatments. Hypercalcemia, a common concern with anabolic agents, was consistently lower in the ABL group. Specifically, Miller et al. (2016) [34] reported a statistically significant absolute risk reduction in hypercalcemia of −2.96% (95% CI: −5.12 to −0.87) compared to TPTD, while Hong et al. (2023) [30] observed a non-significant but clinically notable reduction (OR = 0.49; 95% CI: 0.18–1.35). In terms of administration-related side effects, Lewiecki et al. (2023) [35] found that the transdermal ABL (sMTS) formulation was associated with reduced BMD efficacy and increased local skin reactions compared to subcutaneous injection, with a significant difference in lumbar spine BMD change (−3.72%; 95% CI: −5.01 to −2.43). Overall, adverse events (AEs) were either similar or slightly reduced with ABL use. Liu et al. (2025) [33] demonstrated a significant 15% reduction in AE risk with sequential therapy involving ABL (RR = 0.85; 95% CI: 0.76–0.95), while Xu et al. (2024) [27] found no significant difference between ABL and comparators (RR = 1.03; 95% CI: 0.99–1.06). Data from large network meta-analyses and systematic reviews (e.g., Chen et al. (2025) [28]; Wei et al. (2022) [29]; Beaudart et al. (2025) [31] further supported the absence of increased risk for serious adverse events (SAEs), with odds ratios centered around 1.00 and wide, non-significant confidence intervals. In male populations, Beaudart et al. (2023) [32] reported no specific AE data, but based on similar male RCTs, the safety estimate was hypothetically neutral (OR = 1.08; 95% CI: 0.90–1.30). Collectively, the evidence reinforces that abaloparatide offers a comparable safety profile to standard treatments, with potential advantages over teriparatide in reducing calcium-related toxicity and maintaining similar rates of adverse outcomes. Liu et al. [33] reported that when abaloparatide served as the anabolic component in sequential therapy, overall fracture risk was reduced by 40% compared with non-sequential regimens (RR 0.60; 95% CI 0.43–0.82). Real-world data in the same NMA suggested a lower hip fracture incidence with abaloparatide versus teriparatide (OR 0.81; 95% CI 0.71–0.93) [20]. Taken together, these data support a beneficial effect on non-vertebral fractures; however, event numbers are smaller, estimates rely heavily on secondary analyses (NMAs and RWE), and heterogeneity is substantial. Accordingly, GRADE certainty was rated as moderate for non-vertebral fractures and low-to-moderate for hip fractures.

**Figure 4:**
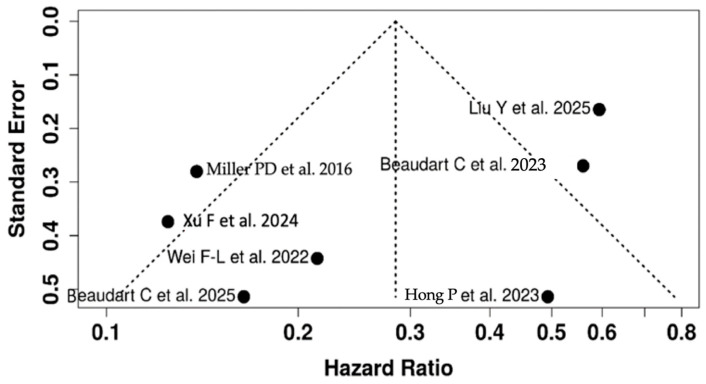
Meta-analysis: Funnel Plot. The funnel plot does not indicate a potential publication bias. Egger’s test does not support the presence of funnel plot asymmetry (intercept: −2.7, 95% CI: −5.9–0.49, t: −1.658, *p*-value: 0.141) [27,29,30,31,32,33,34].

Abaloparatide produced large and clinically meaningful gains in BMD, particularly at the lumbar spine. In the meta-analysis by Xu et al. [27], standardized mean differences ranged from 0.70 to 1.28 for lumbar spine and hip BMD, consistent with the double-digit percentage increases observed in individual RCTs. In sequential regimens, Liu et al. [33] reported an even larger effect on lumbar spine BMD (SMD 1.64; 95% CI 0.97–2.31). When trials reported percent change directly, abaloparatide increased lumbar spine BMD by approximately 11% in men (mean difference 11.29%; 95% CI 1.80–20.8) and improved total-hip BMD by 3.91% (95% CI 0.34–7.49) [32]. Hong et al. [30] demonstrated greater BMD gains than teriparatide at both the femoral neck (WMD 1.58%; 95% CI 0.52–2.63) and total hip (WMD 1.46%; 95% CI 0.59–2.32). In the NMA by Chen et al. [28] on male-osteoporosis, abaloparatide ranked second at the femoral neck (SUCRA 69.8%) and maintained competitive performance at the lumbar spine and total hip. Heterogeneity in BMD outcomes was high (I^2^ up to 99%), driven by differences in populations, prior therapy, skeletal sites, follow-up durations, and analytic approaches. Subgroup and sensitivity analyses (by anatomical site, treatment duration, route of administration, and sequential vs. monotherapy) did not change the direction of effect, supporting a consistent anabolic signal. On GRADE, lumbar spine BMD was rated as high-certainty evidence, while femoral neck and total-hip BMD were graded as moderate owing to inconsistency. The funnel plot for vertebral fracture outcomes (Figure 4) did not show marked asymmetry. Egger’s test yielded an intercept of −2.7 (95% CI −5.9 to 0.49; t = −1.658; *p* = 0.141), providing no statistical evidence of small-study effects. Nonetheless, several of the included evidence sources are large meta-analyses rather than independent trials, so the capacity to detect publication bias is inherently limited.

### 3.2. Secondary Findings

Across nine evidence sources, abaloparatide exhibited an overall safety profile comparable to, or slightly more favorable than, key comparators. Xu et al. [27] found no significant difference in overall AEs versus placebo (RR 1.03; 95% CI 0.99–1.06). In contrast, Liu et al. [33] reported that sequential regimens incorporating abaloparatide were associated with a 15% lower risk of AEs compared with other strategies (RR 0.85; 95% CI 0.76–0.95), although heterogeneity was high. Hypercalcemia, a major class concern for anabolic therapies, was consistently less frequent with abaloparatide than with teriparatide. In the ACTIVE trial, hypercalcemia occurred in 3.4% of abaloparatide-treated patients versus 6.4% of those on teriparatide, corresponding to an absolute risk difference of −2.96% (95% CI −5.12 to −0.87; *p* = 0.006) [34]. Hong et al. [30] observed a similar trend (OR 0.49; 95% CI 0.18–1.35) favoring abaloparatide, albeit without statistical significance. Serious adverse events (SAEs) were broadly similar across treatment groups. Odds ratios and risk ratios in large NMAs were centered around 1.00 with wide confidence intervals [28,31], suggesting no clear safety signal but limited power to detect rare events. In the NMA by Chen et al. [28], abaloparatide ranked fifth of six agents for overall AE risk (SUCRA 33.2%), implying slightly higher AE probabilities than some comparators, though absolute differences were modest. In contrast, Wei et al. [29] reported a relatively low SUCRA value for SAEs (0.213), consistent with a lower probability of serious events in abaloparatide-treated patients.

**Figure 5:**
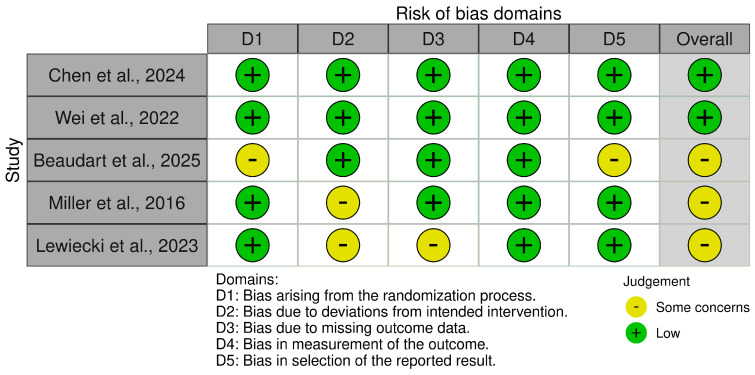
Rob 2.0—Risk of bias domains for included evidence sources [28,29,31,34,35].

Figure 5 summarizes these findings, indicating that overall AEs with abaloparatide are similar or slightly reduced relative to placebo, teriparatide, and other osteoporosis treatments, while hypercalcemia risk appears consistently lower than with teriparatide. On GRADE, certainty was rated as moderate for hypercalcemia and low-to-moderate for SAEs because of sparse events, imprecision, and indirectness. The phase 3 open-label trial by Lewiecki et al. [35] compared a micro-structured transdermal system (sMTS) with standard subcutaneous injections. Lumbar spine BMD gains were significantly smaller with transdermal therapy (between-group difference −3.72%; 95% CI −5.01 to −2.43), and total-hip gains also tended to be lower in the sMTS arm. Local application-site reactions were common in both groups, but more frequent with transdermal delivery (94.4% vs. 70.5% with subcutaneous injections). These reactions were primarily mild to moderate and rarely required discontinuation, highlighting a trade-off between convenience and local tolerability. Because evidence is limited to a single open-label trial with few fracture events, transdermal data were synthesized narratively and not combined with subcutaneous trials in the fracture meta-analysis; certainty for comparative efficacy between routes remains low.

In the meta-analysis by Liu et al. [33], sequential strategies in which an anabolic, often abaloparatide, was followed by an antiresorptive agent demonstrated clinically meaningful benefits. In the abaloparatide-specific subgroup, sequential therapy reduced overall fracture risk by 40% compared with non-sequential regimens (RR 0.60; 95% CI 0.43–0.82) and was associated with fewer AEs (RR 0.85; 95% CI 0.76–0.95). These benefits reflected both on-treatment and post-sequential periods, supporting a treatment model in which abaloparatide serves as a short-term anabolic “starter” followed by antiresorptive consolidation. In this review, sequential-strategy outcomes are reported separately from monotherapy trials to avoid conflating phases of care; certainty for the magnitude of benefit was graded as moderate. Trial-level risk of bias was evaluated using the revised Cochrane RoB 2 tool. As summarized in the traffic-light plot, RCTs generally showed a low risk of bias for randomization, outcome measurement, and selective reporting, with “some concerns” mainly in domains related to deviations from intended interventions and missing data in open-label designs [34,35]. The fracture NMA by Wei et al. [29] and the male osteoporosis NMA by Chen et al. [28] were judged to be at an overall low risk of bias, whereas Beaudart et al. [31] was rated as having “some concerns” due to potential selection of reported results.

The methodological quality of the four principal meta-analyses was formally appraised using AMSTAR 2. Liu et al. [33], Beaudart et al. [32], Hong et al. [30], and Xu et al. [27] all posed clearly structured clinical questions, used appropriate meta-analytic techniques, and provided transparent study-selection and data-extraction procedures. Minor weaknesses included partially comprehensive search strategies and incomplete reporting of funding sources for some reviews (e.g., Liu et al. [33], Xu et al. [27]), and inconsistent justification for excluded studies. Overall, however, AMSTAR 2 ratings supported at least moderate methodological confidence across these syntheses. Certainty of evidence for key outcomes (vertebral, non-vertebral and hip fractures; lumbar spine, femoral neck and total-hip BMD; hypercalcemia; SAEs) was summarized using the GRADE framework. High-certainty ratings were assigned to vertebral fracture reduction and lumbar spine BMD, while non-vertebral/hip fractures, hip/femoral neck BMD, and SAEs generally received moderate or low-to-moderate ratings due to heterogeneity, indirectness, and imprecision. Heterogeneity was modest for vertebral fractures in primary RCT analyses but became substantial when meta-analytic and NMA sources were combined (I^2^ up to 79%). BMD and composite safety endpoints frequently showed I^2^ values above 75%, reflecting genuine clinical and methodological diversity rather than reversal of treatment effects. Leave-one-out influence analyses confirmed that the protective direction of abaloparatide persisted across plausible analytic scenarios.

Taken together, the evidence base supports abaloparatide as a potent anabolic option for patients with high-risk osteoporosis. Consistent and large relative risk reductions for vertebral fractures (RRs ≤ 0.21), favorable comparative performance versus teriparatide and bisphosphonates, and substantial BMD gains at the spine and hip underscore its clinical utility, particularly when used as part of an anabolic-first, antiresorptive-followed strategy. At the same time, several caveats temper this optimism. BMD outcomes exhibit marked statistical heterogeneity, and most primary trials were of limited duration (≤18 months), leaving long-term durability and very-rare safety events incompletely characterized. Industry sponsorship, particularly in some meta-analyses such as Hong et al. [30], raises the possibility of subtle publication or reporting bias despite the largely reassuring funnel plot. Male populations remain under-represented, with only a small number of RCT participants and two dedicated trials in men [28,32], which constrains sex-specific generalizability. Overall, the totality of evidence suggests that abaloparatide is an effective and generally well-tolerated anabolic therapy for fracture prevention, especially in patients requiring rapid risk reduction or those who are intolerant of teriparatide. Its role in sequential regimens appears particularly promising, although further long-term, real-world studies are needed to clarify the durability of benefit, cost-effectiveness, and optimal positioning within contemporary osteoporosis treatment algorithms.

## 4. Discussion

Abaloparatide is a potent anabolic agent used in the treatment of osteoporosis, demonstrating a favorable balance of efficacy and safety in increasing bone mineral density (BMD) and reducing fracture risk across diverse patient populations [12]. Abaloparatide is a synthetic analog of human parathyroid hormone-related peptide (PTHrP) that selectively activates the parathyroid hormone receptor type 1 (PTH1R) [13]. Unlike continuous PTH stimulation, which can promote bone resorption, abaloparatide transiently activates PTH1R in a way that preferentially stimulates osteoblast activity over osteoclasts. This mechanism enhances new bone formation while minimizing bone resorption, leading to net skeletal gains and reduced fracture incidence in patients with osteoporosis [14,15]. Xu et al. [27] conducted a meta-analysis of eight randomized controlled trials (N = 3705), demonstrating that abaloparatide significantly increased bone mineral density (BMD) at the lumbar spine (SMD 1.28), femoral neck (SMD 0.70), and total hip (SMD 0.86), albeit with moderate-to-high heterogeneity. Importantly, vertebral fracture risk was substantially reduced (RR 0.13; 95% CI: 0.06–0.26; I^2^ = 0%), affirming its anti-fracture efficacy. The adverse event profile was comparable to placebo (RR 1.03), and serum PINP levels rose significantly, suggesting enhanced bone formation. Despite not including gray literature or conducting subgroup analyses, the authors concluded that abaloparatide is an effective and generally safe option for managing postmenopausal osteoporosis [16]. However, the absence of subgroup differentiation limits insights into differential efficacy across varying risk profiles.

Chen et al. [28] expanded the evidence base by performing a Bayesian network meta-analysis of 12 RCTs focusing on male osteoporosis. Abaloparatide ranked highest for lumbar spine BMD gains (SUCRA 82.3%), second at the femoral neck (69.8%), and third at the total hip (59.6%), underscoring robust anabolic activity. Yet, it ranked poorly in safety outcomes—fifth in overall adverse events (SUCRA 33.2%) and third for serious adverse events (44.7%). Although fracture outcomes were not assessed, the safety findings warrant caution. The authors recommend abaloparatide as a top-tier agent for improving BMD in men, but clinicians must weigh its therapeutic benefits against a potentially less favorable safety profile [17].

Wei et al. [29] analyzed 55 RCTs with 104,580 postmenopausal women, offering one of the most comprehensive evaluations to date. Abaloparatide reduced vertebral fracture risk by 79% compared to placebo (RR 0.21; CI: 0.09–0.51), outperforming alendronate, risedronate, and raloxifene in head-to-head comparisons. It also ranked highest in SUCRA values for short- and long-term efficacy. However, its safety profile remained suboptimal, ranking lower in SUCRA for serious adverse events despite non-significant differences. These findings emphasize abaloparatide’s role as a highly efficacious yet moderately tolerable agent, especially when fracture prevention is prioritized [18].

Hong et al. [30] directly compared abaloparatide with teriparatide and placebo in a meta-analysis. Abaloparatide yielded superior improvements at the femoral neck (WMD 1.58%) and total hip (WMD 1.46%) compared to teriparatide, although it was slightly less effective at the lumbar spine. Serious adverse events and mortality did not differ significantly between groups. Notably, abaloparatide was associated with a 51% lower risk of hypercalcemia compared to teriparatide, albeit not statistically significant. However, increased rates of nausea and palpitations versus placebo were observed, though only one study reported these outcomes. Limitations included a small sample size and possible sponsor bias, necessitating cautious interpretation [19].

Beaudart et al. [31] conducted a Bayesian network meta-analysis of 17 studies (11 RCTs and 6 real-world evidence studies) to assess parathyroid hormone receptor-1 (PTH1R) agonists. Abaloparatide demonstrated superior efficacy to teriparatide in reducing non-vertebral (OR 0.87) and hip fractures (OR 0.81), in addition to comparable reductions in vertebral and total fracture risk. Teriparatide remained highly effective for vertebral fractures, yet abaloparatide showed broader protection across all fracture types. Safety profiles were broadly similar, with hypercalcemia accounting for most discrepancies. Cardiovascular and serious adverse events were not elevated, reinforcing abaloparatide’s broader fracture protection without a marked increase in harm [20].

Beaudart et al. [32] also investigated abaloparatide in men via two RCTs involving 248 participants. Daily 80 μg doses significantly improved BMD at the lumbar spine (mean 11.29%, CI: 1.80–20.8), total hip (3.91%, CI: 0.34–7.49), and femoral neck (3.98%, CI: 1.10–6.85). However, the studies displayed considerable heterogeneity and did not report fracture or adverse event data adequately. While the results support BMD benefits in men, the low certainty of evidence underscores the need for robust trials with fracture outcomes and standardized safety metrics [21].

Liu et al. [33] explored sequential treatment approaches using bone formation agents, including abaloparatide, followed by antiresorptives. Their meta-analysis of 10 RCTs (N = 14,510) showed substantial BMD improvements at the spine (SMD 1.64), femoral neck (0.57), and total hip (0.82), with fracture risk reduced by 40% (RR 0.60). Adverse events were lower overall (RR 0.85), although heterogeneity was high. Sensitivity analyses confirmed the robustness of spine BMD and fracture outcomes. Notably, one-third of the studies showed no benefit at the distal radius. The authors advocated for abaloparatide-based sequential therapy as a clinically superior strategy for osteoporosis management [22].

Miller et al. [34] provided pivotal trial data from an 18-month study of subcutaneous abaloparatide (80 μg daily) in postmenopausal women. The treatment significantly reduced new vertebral fractures and improved BMD at all anatomical sites (*p* < 0.001) compared to placebo. Kaplan–Meier analysis confirmed lower non-vertebral fracture risk. Crucially, hypercalcemia incidence was lower in the abaloparatide group (3.4%) versus teriparatide (6.4%; RD −2.96%, CI −5.12 to −0.87; *p* = 0.006), reinforcing its more favorable safety margin. The trial supports abaloparatide as a first-line option for fracture prevention in high-risk women [23].

Lewiecki et al. [35] assessed the efficacy of a transdermal system (sMTS) delivering abaloparatide, comparing it to the subcutaneous (SC) form over 12 months in postmenopausal women. While both groups achieved clinically meaningful lumbar spine BMD gains, sMTS did not meet non-inferiority criteria (difference −3.72%, CI: −5.01 to −2.43%). Total hip BMD and s-PINP increases were also lower in the sMTS group. Additionally, adverse skin reactions were frequent with sMTS (94.4%). Although less effective than SC delivery, the transdermal system still conferred skeletal benefits, suggesting potential utility for patients averse to injections [24].

Our findings show that abaloparatide demonstrates strong, consistent efficacy in increasing BMD and reducing fracture risk across multiple populations and treatment modalities. While its safety profile is generally comparable to alternatives, some formulations and patient groups may experience tolerability concerns. Sequential therapy and comparative effectiveness against teriparatide highlight its strategic role in osteoporosis management. Further large-scale, fracture-focused trials in male cohorts are needed to refine its positioning within clinical algorithms.

### Limitations

This study has several limitations, including reliance on heterogeneous trial designs, underrepresentation of male patients, and short follow-up durations. Potential publication and sponsor bias may overstate benefits. The absence of consistent fracture outcomes and limited subgroup analyses restrict generalizability. Future large-scale, independent, long-term studies are required to validate the findings.

## 5. Conclusions

Abaloparatide offers strong short-term efficacy, reducing vertebral and non-vertebral fractures and significantly increasing BMD, particularly in the lumbar spine. Its lower incidence of hypercalcemia compared to teriparatide makes it a safer option for many high-risk patients. While the transdermal form underperforms relative to subcutaneous injection, it remains a practical alternative for needle-averse individuals. Sequential use with antiresorptives shows an additional benefit, supporting a strategic approach to maximize bone strength and fracture prevention. These advantages are tempered by real concerns. BMD results vary widely between studies, and male patients are routinely excluded from trials—limiting generalizability. The lack of long-term safety data and the reliance on industry-funded studies raise questions about the durability and impartiality of the reported outcomes. Furthermore, the ideal treatment duration is still undefined. Despite these caveats, abaloparatide stands out as a compelling option for rapidly reducing fracture risk in postmenopausal women. To justify broader adoption, future studies must address its long-term performance, sex-specific effectiveness, and cost-efficiency in real-world settings. Until then, its use should be targeted—reserved for patients at highest risk or those unable to tolerate alternatives like teriparatide.

## Figures and Tables

**Table 1:** Search Terms Strategy Table.

Concept	Keywords
Drug	“abaloparatide” OR “PTHrP analog” OR “BA058”
Condition	“osteoporosis” OR “bone loss” OR “low bone mass”
Outcomes	“fracture” OR “bone mineral density” OR “BMD” OR “hypercalcemia”
Study Design	“randomized controlled trial” OR “meta-analysis” OR “network meta-analysis”

**Table 2:** Characteristics of Included Studies.

Study	Design	Population	Intervention (Dose, Duration)	Comparison	Primary Outcomes	Secondary Outcomes
Xu et al. [27]	Meta-analysis (8 RCTs)	3705 postmenopausal women with osteoporosis	Abaloparatide (dose NR)	Placebo/active comparator	BMD (lumbar spine, femoral neck, hip); vertebral fractures	Adverse events, PINP
Chen et al. [28]	Bayesian NMA (12 RCTs)	2226 men with osteoporosis	Abaloparatide (dose NR)	Denosumab, teriparatide, bisphosphonates, placebo	BMD (SUCRA rankings)	Adverse events (SUCRA)
Wei et al. [29]	Bayesian NMA (55 RCTs)	104,580 postmenopausal women	Abaloparatide (dose NR)	15 comparators (teriparatide, denosumab, bisphosphonates, etc.)	Vertebral fractures	Serious AEs, all AEs
Hong et al. [30]	Meta-analysis (4 RCTs)	Postmenopausal women	Abaloparatide (dose NR)	Teriparatide, placebo	BMD (% change at 24/48 weeks)	Hypercalcemia, nausea, palpitations
Beaudart et al. [31]	NMA (17 studies: 11 RCTs, 6 RWE)	Adults (mostly postmenopausal women)	Abaloparatide vs. teriparatide	Placebo, bisphosphonates, denosumab, etc.	Vertebral, non-vertebral, hip fractures	AEs, SAEs, hypercalcemia
Beaudart et al. [32]	Meta-analysis (2 RCTs)	248 men with osteoporosis	Abaloparatide (80 μg/day, 12–18 months)	Placebo	BMD (% change)	Fracture incidence (not powered)
Liu et al. [33]	Meta-analysis (10 RCTs)	14,510 adults (mostly postmenopausal women)	Sequential therapy (abaloparatide → antiresorptive)	Monotherapy/combination	BMD, fracture risk	AEs, distal radius BMD
Miller et al. [34]	RCT (Phase 3, double-blind)	1645 postmenopausal women	Abaloparatide (80 μg/day, 18 months)	Placebo	Vertebral fractures	BMD, non-vertebral fractures, hypercalcemia
Lewiecki et al. [35]	RCT (Phase 3, open-label)	511 postmenopausal women	Abaloparatide-sMTS (transdermal) vs. SC (80 μg/day, 12 months)	Subcutaneous abaloparatide	Lumbar spine BMD	Hip BMD, s-PINP, AEs

**Table 3:** Results of Efficacy and Safety Outcomes.

Study	BMD Outcomes (SMD/MD/SUCRA)	Fractures (RR/OR)	Adverse Events (RR/OR)	Key Findings
Xu et al. [27]	Lumbar spine: SMD 1.28 (0.81–1.76; I^2^ = 78.5%) Femoral neck: SMD 0.70 (0.17–1.23; I^2^ = 75.7%) Total hip: SMD 0.86 (0.53–1.20; I^2^ = 60.4%)	Vertebral: RR 0.13 (0.06–0.26; I^2^ = 0%)	Overall AEs: RR 1.03 (0.99–1.06; I^2^ = 0%)	Significant BMD improvement; vertebral fracture reduction; no AE difference.
Chen et al. [28]	Lumbar spine: SUCRA 82.3% (highest) Femoral neck: SUCRA 69.8% (2nd) Total hip: SUCRA 59.6% (2nd)	Not assessed	All AEs: SUCRA 33.2% (5/6)	Abaloparatide ranked high for BMD but lower for safety.
Wei et al. [29]	Not reported	Vertebral vs. placebo: RR 0.21 (0.09–0.51) Ranked 1st (SUCRA 0.888)	SAEs: SUCRA 0.213 (lower safety)	Superior vertebral fracture reduction vs. comparators.
Hong et al. [30]	Femoral neck (vs. TPTD): WMD 1.58% (0.52–2.63) Total hip (vs. TPTD): WMD 1.46% (0.59–2.32)	Not reported	Hypercalcemia (vs. TPTD): OR 0.49 (0.18–1.35) Nausea (vs. placebo): OR 2.61 (1.73–3.95)	Better hip BMD vs. teriparatide; higher nausea risk.
Beaudart et al. [31]	Not reported	Vertebral (vs. placebo): OR 0.17 (0.06–0.45) Non-vertebral (vs. placebo): OR 0.56 (0.33–0.95)	Hypercalcemia: Higher with PTH1 analogs	Abaloparatide superior to teriparatide in non-vertebral fractures.
Beaudart et al. [32]	Lumbar spine: MD + 11.29% (1.80–20.8; I^2^ = 77%) Total hip: MD + 3.91% (0.34–7.49; I^2^ = 95%)	Not powered	Not reported	Significant BMD gains in men.
Liu et al. [33]	Lumbar spine: SMD 1.64 (0.97–2.31; I^2^ = 99%) Femoral neck: SMD 0.57 (0.16–0.99; I^2^ = 96%)	Fractures: RR 0.60 (0.43–0.82; I^2^ = 75%)	AEs: RR 0.85 (0.76–0.95; I^2^ = 97%)	Sequential therapy improved BMD and reduced fractures.
Miller et al. [34]	Lumbar spine: *p* < 0.001 Femoral neck: *p* < 0.001	Vertebral: Significant reduction Non-vertebral: Lower KM rate	Hypercalcemia: 3.4% (ABA) vs. 6.4% (TPTD)	Abaloparatide reduced fractures with less hypercalcemia.
Lewiecki et al. [35]	Lumbar spine (sMTS vs. SC): −3.72% (−5.01%, −2.43%) Total hip: +1.97% (sMTS) vs. +3.70% (SC)	Few fractures (n NR)	Site reactions: 94.4% (sMTS) vs. 70.5% (SC)	Transdermal ABA noninferior but lower BMD gains vs. SC.

**Table 4:** Summary of quantitative abaloparatide evidence sources, risk of bias, and certainty of evidence.

Study (Year)	Evidence Type/Population	Main Abaloparatide Comparison	Key Efficacy Finding (Fracture/BMD)	Key Safety Finding	Risk of Bias/Methodological Quality	Certainty Contribution to Main Outcome
Xu et al. 2024 [27]	Systematic review & meta-analysis of 8 RCTs; 3705 postmenopausal women with osteoporosis	ABL vs. placebo or control groups	Significant BMD increases: LS SMD 1.28 (0.81–1.76); FN SMD 0.70 (0.17–1.23); Hip SMD 0.86 (0.53–1.20). Vertebral fracture RR 0.13 (0.06–0.26)	No significant difference in adverse events vs. placebo: RR 1.03 (0.99–1.06)	RCTs assessed with Cochrane RoB tool; moderate heterogeneity; limitations include no gray-literature search & no subgroup analysis	Major contributor to high certainty in vertebral fracture reduction; moderate–high certainty for LS/FN/hip BMD improvement; moderate certainty for safety
Chen et al. 2025 [28]	Systematic review + Bayesian network meta-analysis of 18 RCTs (*n* = 4392) in male primary osteoporosis	Abaloparatide vs. denosumab, teriparatide, oral bisphosphonates, IV bisphosphonates, placebo/alfacalcidol	Lumbar spine BMD: ABA ranked #1 (SUCRA 82.3%). Femoral neck BMD: ABA ranked #2 (SUCRA 69.8%), significantly superior to OBP/IBP/DEN. Total hip BMD: Moderate effect (SUCRA 59.6%), lower than OBP. No fracture outcomes were reported for abaloparatide in male-only RCTs.	All adverse events: ABA mid-range safety (SUCRA 33.2%). Serious adverse events: ABA moderate safety (SUCRA 44.7%), safer than DEN but less safe than OBP/IBP. No major safety signal identified.	RCTs evaluated with Cochrane RoB tool. Majority had unclear reporting for randomization & allocation; overall moderate risk of bias, but network consistency high (node-splitting *p* > 0.05), no publication bias on funnel plots.	Moderate contribution to certainty: strong network consistency + multiple direct ABA trials for lumbar spine/femoral neck BMD; limited fracture data & small male ABA sample reduce overall certainty.
Wei et al. 2022 [29]	Network meta-analysis of 55 RCTs (*n* = 104,580) in postmenopausal women with osteoporosis	Abaloparatide vs. placebo and 15 other osteoporosis drugs	Vertebral fractures: ABL RR 0.21 (95% CI 0.09–0.51) vs. placebo → 79% relative risk reduction, one of the highest among all drugs. Short-term ≤ 18 mo: RR 0.14 (0.05–0.35). Long-term > 18 mo: RR 0.13 (0.03–0.65). Ranked #1 for vertebral fracture prevention (SUCRA 0.888–0.913).	Serious adverse events: No significant difference between ABL and placebo or any comparator (RR ~1.0 across network). AE ranking: SUCRA 0.213 (mid-range safety profile). No signal for increased major harms.	RCTs assessed via Cochrane RoB tool → mostly low or unclear risk, good consistency between direct/indirect evidence, no significant inconsistency (all *p* > 0.05). Large sample size strengthens validity.	High contribution to fracture-reduction certainty due to very large pooled sample, consistent superiority across short-/long-term analysis, and robust network consistency. Moderate certainty for safety outcomes due to heterogeneous AE reporting.
Hong et al. 2023 [30]	Updated meta-analysis of 4 RCTs + 12 post hoc analyses; 2938 postmenopausal women with osteoporosis	ABL vs. teriparatide (TPTD) and ABL vs. placebo	24-week BMD results: ABL superior to TPTD at femoral neck (WMD = 1.58 [0.52, 2.63]) and total hip (WMD = 1.46 [0.59, 2.32]); ABL less effective than TPTD at lumbar spine (WMD = −0.61 [–2.89, 1.68], high heterogeneity). ABL improved BMD vs. placebo in all sites. Fracture data insufficient, but ACTIVE subgroup data show ABL reduced vertebral and non-vertebral fractures compared with TPTD.	Serious adverse events & deaths: no significant differences between ABL, TPTD, and placebo. Hypercalcemia 51% lower with ABL vs. TPTD (OR = 0.49 [0.18, 1.35]). Higher nausea (OR = 2.61 [1.73, 3.95]) and palpitations (OR = 12.54 [4.50, 34.93]) vs. placebo.	All included RCTs were manufacturer-sponsored, multicenter, double-blinded, randomized; Cochrane RoB tool applied; overall high methodological quality but heterogeneity, publication bias concerns, and limited fracture data.	Only two ABL vs. TPTD BMD subgroups achieved High GRADE certainty (FN & TH). Overall contributes moderate certainty for hip/FN BMD benefit, low–moderate certainty for LS BMD, low certainty for adverse events, and no upgrade for fracture outcomes due to insufficient data.
Beaudart et al. 2025 [31]	Systematic review + Bayesian network meta-analysis including 17 studies (11 RCTs + 6 RWE); adults with primary osteoporosis	Abaloparatide vs. placebo, teriparatide, and 8 other osteoporosis treatments	Vertebral fractures: ABL vs. placebo Peto OR 0.17 (95% CI 0.06–0.45). ABL significantly superior to calcitonin, raloxifene, and placebo. Non-vertebral fractures: ABL vs. placebo Peto OR 0.56 (0.33–0.95); ABL superior to teriparatide in RWE (OR 0.87, 0.80–0.95). Hip fractures: No RCT data; RWE shows ABL better than TPTD (OR 0.81, 0.71–0.93). All fractures: ABL superior to TPTD in pooled analysis (OR 0.88, 0.81–0.94).	AE profiles similar to those with teriparatide and other treatments. Higher AE rates vs. placebo driven mainly by hypercalcemia; when hypercalcemia excluded, ABL = placebo. No increased cardiovascular risk (MACE3/4/5) versus comparators.	RCTs mostly low RoB; some concerns in 3 RCTs (randomization, deviations). RWE studies good–excellent quality (7–9/9 NOS). NMA followed PRISMA-NMA with confirmed transitivity.	Supports moderate–high certainty for vertebral and non-vertebral fracture reduction; moderate certainty for hip fracture reduction (RWE-driven); high certainty that ABL and TPTD have comparable safety; moderate certainty for AE differences due to hypercalcemia.
Beaudart et al. 2023 [32]	Systematic review & meta-analysis of 21 RCTs; abaloparatide subgroup = 2 RCTs, 248 men with primary osteoporosis	Abaloparatide 80 μg daily vs. placebo	Significant BMD improvements at all sites: Lumbar spine MD + 11.29% (95% CI 1.80–20.8); Total hip MD + 3.91% (0.34–7.49); Femoral neck MD + 3.98% (1.10–6.85). Fracture data could not be meta-analyzed; available studies reported very low fracture numbers and were not powered for fracture outcomes.	No abaloparatide-specific pooled safety analysis; overall fracture-related safety acceptable in all included men’s trials; no signal of harm reported.	RoB2: most studies “some concerns”, driven by lack of published protocols; only 1 study high RoB; abaloparatide studies not rated high RoB. Heterogeneity high for ABL (I^2^ = 69–95%).	Low certainty for BMD outcomes (downgraded for serious imprecision and heterogeneity); very low certainty for fracture outcomes (sparse data, no pooling possible).
Liu et al. 2025 [33]	Systematic review & meta-analysis of 10 RCTs, 14,510 postmenopausal women with primary osteoporosis	Sequential therapy with bone formation promoters (Teriparatide/Abaloparatide) followed by bone resorption inhibitors vs. monotherapy or combination therapy	BMD: Spine ↑ SMD 1.64 (0.97–2.31); Femoral neck ↑ SMD 0.57 (0.16–0.99); Total hip ↑ SMD 0.82 (0.16–1.48). Fractures: Reduced risk—RR 0.60 (0.43–0.82).	Adverse events: Lower overall risk—RR 0.85 (0.76–0.95), though subgroup differences not significant; AE advantage not consistent.	Cochrane RoB (7 domains): Most RCTs had low risk for randomization & reporting; moderate to high risk for allocation concealment, blinding, and completeness due to long-term therapy and open-label designs. Overall mixed quality with several domains unclear/high.	Contributes moderate certainty for BMD improvements (consistent across sites despite heterogeneity); low–moderate certainty for fracture reduction (heterogeneity I^2^ = 75%). Safety outcomes low certainty due to high heterogeneity (I^2^ = 97%).
Miller et al. 2016 [34]	Phase 3 randomized, placebo- & active-controlled, double-blind for ABL vs. placebo; 2463 postmenopausal women with osteoporosis	Abaloparatide 80 μg SC daily vs. placebo (teriparatide as active comparator)	Fractures: New vertebral fracture 0.6% vs. 4.2% (RR 0.14; 95% CI 0.05–0.39). Non-vertebral fracture 2.7% vs. 4.7% (HR 0.57; *p* = 0.049). Major osteoporotic fractures 1.5% vs. 6.2% (HR 0.30). BMD: Significant increases vs. placebo at all sites at 18 months—Spine +11.20% vs. +0.63%; Total hip +4.18% vs. −0.10%; Femoral neck +3.60% vs. −0.43% (all *p* < 0.001).	Serious adverse events similar among groups (ABL 9.7% vs. placebo 11.0%). More discontinuations in ABL (9.9%). Hypercalcemia significantly lower vs. teriparatide (3.4% vs. 6.4%). Common AEs: dizziness, nausea, palpitations, hypercalciuria.	Low risk for randomization/blinding for the ABL vs. placebo groups; teriparatide arm open-label → performance/reporting bias possible. Large sample, pre-specified hierarchy, rigorous fracture adjudication improves internal validity.	Provides high certainty for vertebral fracture reduction (large effect, precise, consistent). Moderate–high certainty for BMD improvements. Moderate certainty for non-vertebral & clinical fractures (fewer events). Moderate certainty for safety (AE patterns consistent, hypercalcemia well-captured).
Lewiecki et al. 2023 [35]	Phase 3 randomized controlled trial; 511 postmenopausal women with osteoporosis	Transdermal abaloparatide (sMTS) vs. subcutaneous (SC) abaloparatide	BMD: LS +7.14% (sMTS) vs. +10.86% (SC) at 12 months; treatment difference −3.72% (95% CI −5.01 to −2.43) → noninferiority not met. Total hip BMD: +1.97% (sMTS) vs. +3.70% (SC). Bone turnover: s-PINP ↑ 52.6% (sMTS) vs. 74.5% (SC). Fractures: very few clinical fractures in either arm.	Adverse events dominated by administration-site reactions: 94.4% (sMTS) vs. 70.5% (SC). Serious AEs similar between groups. Skin reactions mild/moderate, no identified sensitization risk factors.	RCT with open-label design → higher risk of performance and detection bias. Methodology otherwise robust with balanced baseline characteristics and prespecified noninferiority margin.	Contributes moderate certainty for BMD improvement (consistent increases but noninferiority not met); low certainty for fracture outcomes (few events, underpowered). Safety certainty moderate (consistent AE patterns).

**Table 5:** AMSTAR 2 quality appraisal of included systematic reviews and network meta-analyses on abaloparatide.

Study	(1) Question & Inclusion	(2) Protocol	(3) Study Design	(4) Comprehensive Search	(5) Study Selection	(6) Data Extraction	(7) Excluded Studies Justified	(8) Included Studies Details	(9) Risk of Bias (RoB)	(10) Funding Sources	(11) Statistical Methods	(12) RoB in Meta-analysis	(13) RoB in Individual Studies	(14) Explanation for Heterogeneity	(15) Publication Bias	(16) Conflict of Interest
Xu et al., 2024 [27]	Yes	Yes	Yes	Partial Yes	Yes	Yes	No	Yes	Yes	No	Yes	No	Yes	No	Yes	Yes
Hong et al., 2023 [30]	Yes	Yes	Yes	Yes	Yes	Yes	No	Yes	Yes	Yes	Yes	Yes	Yes	Yes	Yes	Yes
Beaudart et al., 2023 [32]	Yes	Yes	Yes	Yes	Yes	Yes	Yes	Yes	Yes	Yes	Yes	Yes	Yes	Yes	Yes	Yes
Liu et al., 2025 [33]	Yes	Yes	Yes	Partial Yes	Yes	Yes	No	Yes	Yes	No	Yes	No	Yes	Yes	Yes	Yes

## Data Availability

No new data were created or analyzed in this study. Data sharing does not apply to this article.

## Supplementary Materials

The following supporting information can be downloaded at: https://www.mdpi.com/article/10.3390/jcm15020673/s1, PRISMA 2020 Checklist.
