# Supplementary material for: The Efficacy and Safety of Abaloparatide in Osteoporosis: A Systematic Review and Meta-Analysis
Source: J Clin Med. 2026 Jan 14;15(2):673. doi: 10.3390/jcm15020673 (PMC12842433; doi:10.3390/jcm15020673)
Supplement: Supplementary file 1 [file jcm-15-00673-s001.zip › jcm-3951288-supplementary.pdf]

PRISMA 2020 Checklist

Section and Topic	Item #	Checklist Item	Location in Manuscript (Where Item is Reported)
TITLE			
Title	1	Identify the report as a systematic review.	**Title:** "Efficacy and Safety of Abaloparatide in Osteoporosis: A Systematic Review and Meta-Analysis"
ABSTRACT			
Abstract	2	See the PRISMA 2020 for Abstracts checklist.	**Abstract:** The entire abstract is structured with Aim, Methodology, Results, and Conclusion.
INTRODUCTION			
Rationale	3	Describe the rationale for the review in the context of existing knowledge.	**Introduction, Paragraph 4:** "However, anabolic use is limited by cost, strict treatment duration, and insufficient comparative data... existing reviews inadequately address abaloparatide's clinical positioning, benefit-risk profile, and comparative efficacy [10,11]."
Objectives	4	Provide an explicit statement of the objective(s) or question(s) the review addresses.	**Abstract (Aim):** "evaluates the efficacy and safety of abaloparatide..."<br>**Introduction, Final Sentence:** "This meta-analysis was designed to address these critical gaps... aiming to provide a more granular understanding of abaloparatide's therapeutic value..."
METHODS			
Eligibility criteria	5	Specify the inclusion and exclusion criteria for the review and how studies were grouped for the syntheses.	**Material and Methods (Eligibility Criteria):** The PICOS table with detailed Inclusion and Exclusion Criteria.
Information sources	6	Specify all databases, registers, websites, organisations, reference lists and other sources searched. Specify the date when each source was last searched or consulted.	**Material and Methods (Search Strategy and Data Sources):** "PubMed, Embase, Cochrane CENTRAL, and Web of Science... manual reference checks... search was last updated in January 2025."
Search strategy	7	Present the full search strategies for all databases, registers and websites, including any filters and limits used.	**Material and Methods (Search Strategy and Data Sources):** The "Search Terms Strategy Table" listing concepts and keywords. The date limit "2016–2024" is also specified.
Selection process	8	Specify the methods used to decide whether a study met the inclusion criteria, including how many reviewers screened each record and each report retrieved, whether they worked independently, and if applicable, details of automation tools used in the process.	**Material and Methods (Data Extraction and Synthesis):** "Two reviewers independently extracted data... Discrepancies were resolved by discussion or third-party arbitration."
Data collection process	9	Specify the methods used to collect data from reports, including how many reviewers collected data from each report, whether they worked independently, any processes for obtaining or confirming data from study investigators, and if applicable, details of automation tools used in the process.	**Material and Methods (Data Extraction and Synthesis):** "Two reviewers independently extracted data using a pre-specified form... Discrepancies were resolved by discussion or third-party arbitration."
Data items	10a	List and define all outcomes for which data were sought. Specify whether all results that were compatible with each outcome domain in each study were sought (e.g. for all measures, time points, analyses), and if not, the methods used to decide which results to collect.	**Material and Methods (Outcomes Assessed):** "Primary outcomes included changes in BMD... and incidence of vertebral, non-vertebral, and hip fractures. Secondary outcomes included incidence of hypercalcemia, all adverse events..."
	10b	List and define all other variables for which data were sought (e.g. participant and intervention characteristics, funding sources). Describe any assumptions made about any missing or unclear information.	**Material and Methods (Data Extraction and Synthesis):** The form captured "study design, population characteristics, intervention regimen (dose, duration, delivery), comparators, primary and secondary outcomes, and funding sources."
Study risk of bias assessment	11	Specify the methods used to assess risk of bias in the included studies, including details of the tool(s) used, how many reviewers assessed each study and whether they worked independently, and if applicable, details of automation tools used in the process.	**Abstract (Methodology):** "Risk of bias was assessed via Cochrane RoB 2.0 and GRADE."
Effect measures	12	Specify for each outcome the effect measure(s) (e.g. risk ratio, mean difference) used in the synthesis or presentation of results.	**Material and Methods (Data Extraction and Synthesis):** "continuous outcomes... summarized as standardized mean differences (SMD) or weighted mean differences (WMD)... binary outcomes... as risk ratios (RR) or odds ratios (OR)"

Synthesis methods	13a	Describe the processes used to decide which studies were eligible for each synthesis (e.g. tabulating the study intervention characteristics and comparing against the planned groups for each synthesis (item #5)).	Implied by the PICOS framework and synthesis methods described.
	13b	Describe any methods required to prepare the data for presentation or synthesis, such as handling of missing summary statistics, or data conversions.	Not explicitly described in the provided text.
	13c	Describe any methods used to tabulate or visually display results of individual studies and syntheses.	**Material and Methods (Methodological Summary):** "Comprehensive quantitative synthesis". **Results:** Tables and figures (forest plots) are presented.
	13d	Describe any methods used to synthesize results and provide a rationale for the choice(s). If meta-analysis was performed, describe the model(s), method(s) to identify the presence and extent of statistical heterogeneity, and software package(s) used.	**Material and Methods (Data Extraction and Synthesis):** "Meta-analyses were conducted using random-effects models due to expected clinical heterogeneity. Heterogeneity was assessed via the I^2 statistic... and Cochrane's Q-test."
	13e	Describe any methods used to explore possible causes of heterogeneity among study results (e.g. subgroup analysis, meta-regression).	**Material and Methods (Data Extraction and Synthesis):** "Subgroup analyses were performed by sex, delivery method (transdermal vs. subcutaneous), and treatment strategy (monotherapy vs. sequential)."
	13f	Describe any sensitivity analyses conducted to assess robustness of the synthesized results.	Not explicitly described in the provided text.
Reporting bias assessment	14	Describe any methods used to assess risk of bias due to missing results in a synthesis (arising from reporting biases).	**Meta-analysis section (Funnel Plot Data Table):** "The Egger's test does not support the presence of funnel plot asymmetry..."
Certainty assessment	15	Describe any methods used to assess certainty (or confidence) in the body of evidence for an outcome.	**Abstract (Methodology):** "GRADE".
RESULTS			
Study selection	16a	Describe the results of the search and selection process, from the number of records identified in the search to the number of studies included in the review, ideally using a flow diagram.	**Figure 1:** "Prisma Flow Diagram of Included PAPERS"
	16b	Cite studies that might appear to meet the inclusion criteria, but which were excluded, and explain why they were excluded.	Not provided in the text. Typically included in the flow diagram.
Study characteristics	17	Cite each included study and present its characteristics.	**Table 1: "Characteristics of Included Studies"** lists each study (Xu, Chen, Wei, etc.) and their design, population, intervention, and outcomes.
Risk of bias in studies	18	Present assessments of risk of bias for each included study.	Not presented in a summary table or figure for individual studies. Mentioned narratively in **Limitations and Bias Considerations**.
Results of individual studies	19	For all outcomes, present, for each study: (a) summary statistics for each group (where appropriate) and (b) an effect estimate and its precision (e.g. confidence/credible interval), ideally using structured tables or plots.	**Table 2: "Results of Efficacy and Safety Outcomes"** provides effect estimates (SMD, RR, OR, SUCRA) and 95% CIs for each study.
Results of syntheses	20a	For each synthesis, briefly summarise the characteristics and risk of bias among contributing studies.	Not explicitly done for each synthesis. General comments are in **Limitations and Bias Considerations**.
	20b	Present results of all statistical syntheses conducted. If meta-analysis was done, present for each the summary estimate and its precision (e.g. confidence/credible interval) and measures of statistical heterogeneity. If comparing groups, describe the direction of the effect.	**Primary Findings and Secondary Findings** sections narratively describe results. **Table 2** provides study-level data. **Figures 3, 4, 5, 6** show forest plots with pooled estimates and heterogeneity (I^2).
	20c	Present results of all investigations of possible causes of heterogeneity among study results.	**Primary/Secondary Findings:** High heterogeneity (e.g., I^2=99%) is reported and attributed to "protocol differences and short trial durations".
	20d	Present results of all sensitivity analyses conducted to assess the robustness of the synthesized results.	Not presented.
Reporting biases	21	Present assessments of risk of bias due to missing results (arising from reporting biases) for each synthesis assessed.	**Meta-analysis section (Funnel Plot Data Table):** "The funnel plot does not indicate a potential publication bias. The Egger's test... p-value: 0.141."
Certainty of evidence	22	Present assessments of certainty (or confidence) in the body of evidence for each outcome assessed.	Not presented in a summary table (e.g., GRADE profile). Discussed narratively in **Critical Interpretation** (e.g., "high-confidence assessment").
DISCUSSION			

Discussion	23a	Provide a general interpretation of the results in the context of other evidence.	**Critical Interpretation, 1st Paragraph:** "The evidence base supports abaloparatide as a potent anabolic therapy..."
	23b	Discuss any limitations of the evidence included in the review.	**Critical Interpretation, 2nd Paragraph:** "However, some limitations temper this optimism... high heterogeneity... industry sponsorship... Long-term efficacy and safety remain uncertain... underrepresentation of men..."
	23c	Discuss any limitations of the review processes used.	**Limitations and Bias Considerations:** "high statistical heterogeneity (I^2 up to 99%)... Industry sponsorship in some included meta-analyses... introduced potential bias."
	23d	Discuss implications of the results for practice, policy, and future research.	**Critical Interpretation, Final Paragraph:** "Abaloparatide offers a compelling option for high-risk osteoporosis patients... Its use in sequential therapy regimens may be a key strategy... long-term data... are needed to validate its broader adoption..."
OTHER INFORMATION			
Registration and protocol	24a	Provide registration information for the review, including register name and registration number, or state that the review was not registered.	**Material and Methods (Study Design and Registration):** "pre-registered in PROSPERO (Registration ID: if any put here)". *[Note: Placeholder text used]*
	24b	Indicate where the review protocol can be accessed, or state that a protocol was not prepared.	Not provided.
	24c	Describe and explain any amendments to information provided at registration or in the protocol.	Not provided.
Support	25	Describe sources of financial or non-financial support for the review, and the role of the funders or sponsors in the review.	Not provided.
Competing interests	26	Declare any competing interests of review authors.	Not provided.
Availability of data, code and other materials	27	Report which of the following are publicly available and where they can be found: template data collection forms; data extracted from included studies; data used for all analyses; analytic code; any other materials used in the review.	Not provided.
